# A Proline Racemase Based PCR for Identification of *Trypanosoma vivax* in Cattle Blood

**DOI:** 10.1371/journal.pone.0084819

**Published:** 2014-01-08

**Authors:** Regassa Fikru, Ashenafi Hagos, Stijn Rogé, Armando Reyna-Bello, Mary Isabel Gonzatti, Bekana Merga, Bruno Maria Goddeeris, Philippe Büscher

**Affiliations:** 1 College of Veterinary Medicine and Agriculture, Addis Ababa University, Debre Zeit, Ethiopia; 2 Department of Biomedical Sciences, Institute of Tropical Medicine, Antwerp, Belgium; 3 Department Biosystems, Faculty of Bioscience Engineering, Katholieke Universiteit Leuven, Leuven, Belgium; 4 Grupo de Inmunobiología, Centro de Estudios Biomédicos y Veterinarios, Universidad acional Experimental Simón Rodríguez, Caracas, Venezuela; 5 Grupo de Bioquímica e Inmunología de Hemoparásitos, Departamento de Biología Celular, Universidad Simón Bolívar, Caracas, Venezuela; Obihiro University of Agriculture and Veterinary Medicine, Japan

## Abstract

A study was conducted to develop a *Trypanosoma vivax (T. vivax)* specific PCR based on the *T. vivax* proline racemase (*Tv*PRAC) gene. Forward and reverse primers were designed that bind at 764–783 bp and 983–1002 bp of the gene. To assess its specificity, *Tv*PRAC PCR was conducted on DNA extracted from different haemotropic pathogens: *T. vivax* from Nigeria, Ethiopia and Venezuela, *T. congolense* Savannah type, *T. brucei brucei*, *T. evansi*, *T. equiperdum*, *T. theileri*, *Theileria parva*, *Anaplasma marginale*, *Babesia bovis* and *Babesia bigemina* and from bovine, goat, mouse, camel and human blood. The analytical sensitivity of the *Tv*PRAC PCR was compared with that of the ITS-1 PCR and the 18S PCR-RFLP on a dilution series of *T. vivax* DNA in water. The diagnostic performance of the three PCRs was compared on 411 Ethiopian bovine blood specimens collected in a former study. *Tv*PRAC PCR proved to be fully specific for *T. vivax*, irrespective of its geographical origin. Its analytical sensitivity was lower than that of ITS-1 PCR. On these bovine specimens, *Tv*PRAC PCR detected 8.3% *T. vivax* infections while ITS-1 PCR and 18S PCR-RFLP detected respectively 22.6 and 6.1% *T. vivax* infections. The study demonstrates that a proline racemase based PCR could be used, preferably in combination with ITS-1 PCR, as a species-specific diagnostic test for *T. vivax* infections worldwide.

## Introduction

Trypanosomoses are diseases caused by species of the genus *Trypanosoma*. Within the Salivarian trypanosomes, *Trypanosoma vivax*, *Trypanosoma congolense* and *Trypansoma brucei* are the three most important pathogenic species in livestock responsible for considerable production losses and morbidity. Two subspecies of *T. brucei*, i.e. *T.b. gambiense* and *T.b. rhodesiense* cause human African trypanosomosis (sleeping sickness). Animal trypanosomosis caused by *T. vivax* is a widespread disease in Africa and South America, hindering livestock production and food self-sufficiency [1]–[3]. Compared to standard parasitological techniques, molecular diagnostic tools, and in particular the polymerase chain reaction, allow the detection of trypanosome infections with much lower parasite numbers, both in the vertebrate and in the insect host [4]–[7]. The diversity of PCR methods for identification of trypanosome taxa has also led to increased appreciation of trypanosome genetic diversity. For identification of the trypanosome species in biological specimens from mammal or insect hosts, ribosomal genes and internal transcribed spacers are often chosen as target sequences [4], [7]–[10]. For *T. vivax*, also other target sequences have been used such as satellite and microsatellite sequences, spliced-leader sequences and cathepsin L-like genes [6], [11]–[13]. In some studies it was shown that the DNA sequences initially used as *T. vivax* specific target for hybridisation or for PCR primers, were only specific for the West African form of *T. vivax* that also occurs in South America [2], [6], [14], [15] [16]. However, in East Africa, other *T. vivax* genotypes circulate [17]–[20]. In a study, carried out in Ethiopia, on bovine trypanosomosis [21] we observed that results obtained with 18S PCR [22] and TVM PCR [23] were not consistent with ITS-1 PCR [4] with respect to *T. vivax* detection. In order to unequivocally identify the infecting trypanosome species, we developed a PCR targeting the proline racemase (PRAC) gene. The PRAC gene, originally described for *T. cruzi*, was also found in the genome of the West African *T. vivax* ILRAD 1392 strain isolated in Nigeria and was shown to be absent in other African trypanosomes [24]. In *T. cruzi*, PRAC plays a crucial role in the interconversion of free L- and D-proline enantiomers. The biochemical characterisation of the corresponding protein revealed that *T. vivax* proline racemase (*Tv*PRAC) exhibits characteristics and kinetic parameters comparable to *T. cruzi* PRAC [24]. We hereby report on the application of *Tv*PRAC PCR to discriminate *T. vivax* from the other trypanosome taxa.

## Materials and Methods

The sequence of the *Tv*PRAC gene (1038 bp) from *T.vivax* ILRAD 1392 was obtained from GenBank using accession number EF175213.1. We designed primers that specifically annealed to *T. vivax* DNA and not to DNA from other *Kinetoplastida*, *Bovidae*, *Hominoidea* and *Mus*. *Tv*PRAC-F forward primer 5′CGCAAGTGGACCGTTCGCCT3′ and *Tv*PRAC-R reverse primer 5′ACGCGGGGCGAACAGAAGTG 3′ are complementary to bp 764–783 and 983–1002 of *Tv*PRAC and generate an amplicon with an expected length of 239 bp only in *T. vivax* and not in *T. cruzi*. The primer binding sites encompass the MIII* PRAC motif and R3 (tyrosine) residue of the C-terminal half of the protein [24]. After optimisation, the following PCR protocol was retained: a 25 µL reaction volume contained 2.5 mM MgCl_2_ (Qiagen), 200 µM of each dNTP (Eurogentec), 0.8 µM of *Tv*PRAC-F and *Tv*PRAC-R primers (Biolegio), 0.5 U Hotstart *Taq* polymerase (Qiagen,) 0.1 mg/mL acetylated BSA (Promega) and 2.5 µL target DNA. To assess the analytical sensitivity of *Tv*PRAC PCR, a Hotstart master mix (Qiagen) was used with 1.5 mM MgCl_2_ and without acetylated BSA. Cycling conditions were: 94°C for 15 min, 40 cycles of 30 sec at 94°C followed by 30 sec at 63°C and 30 sec at 72°C, final extension at 72°C for 5 min. The PCR product was electrophorised in a 2% agarose gel and stained with ethidium bromide (EtBr) for detection of the specific 239 bp band. The *Tv*PRAC PCR products were either directly sequenced using both forward and reverse *Tv*PRAC primers or sequenced after cloning in a pCR®4-TOPO vector followed by transformation into TOP10 chemically competent *E.coli*, as described in the TOPO® TA Cloning® Kit for Sequencing(Invitrogen). Sequencing was carried out at the VIB facilities at the Antwerp University. The obtained sequences were aligned to each other using CLC Sequence Viewer 6 and compared with the sequence of the *Tv*PRAC gene of ILRAD 1392.

For assaying specificity of the *Tv*PRAC PCR, DNA was prepared from bovine, goat, camel, mouse and human blood, and from different trypanosome species and other pathogens affecting cattle (**Table 1**). DNA from *T. vivax* Sh, *Anaplasma marginale*, *Babesia bovis* and *Babesia bigemina* were extracted from blood of an infected calf. DNA from *T. vivax* LIEM 176 was extracted from blood of infected sheep. Therefore, the concentration of *T. vivax* DNA in these specimens remains unknown. DNA from the other pathogen strains was extracted from purified cells. To detect any contamination during extraction, a sample of DNA-free H_2_0 ( =  negative extraction control) was processed along with the other specimens. The species identity of the trypanosomes included in this study was confirmed by ITS-1 PCR that yields taxon specific amplicon lengths of 150 bp, 350 bp, 450 bp and 650 bp, for *T. vivax*, *T. theileiri*, *Trypanozoon* and *T. congolense* Savannah type, respectively.

**Table 1:** Pathogen strains used to extract DNA for development and evaluation of *Tv*PRAC PCR.

Taxon	Name	Reference	Origin
*Trypanosoma vivax*	Y486	[26]	Nigeria
*Trypanosoma vivax*	MBOV/ET/2012/AAU-CVMA/001, alias 4337	Unpublished	Ethiopia a
*Trypanosoma vivax*	MBOV/ET/2012/AAU-CVMA/002, alias 4338	Unpublished	Ethiopia a
*Trypanosoma vivax*	MBOV/ET/2012/AAU-CVMA/003, alias Di	Unpublished	Ethiopia a
*Trypanosoma vivax*	MBOV/ET/2012/AAU-CVMA/004, alias Fc	Unpublished	Ethiopia b
*Trypanosoma vivax* c	MBOV/ET/2012/AAU-CVMA/005, alias Sh	Unpublished	Ethiopia b
*Trypanosoma vivax* d	LIEM 176	[15]	Venezuela
*Trypanosoma congolense*	TRT57 (Savannah type)	[27]	Zambia
*Trypanosoma brucei brucei*	AnTar 1	[28]	Uganda
*Trypanosoma evansi*	RoTat 1.2	[29]	Indonesia
*Trypanosoma equiperdum*	OVI	[30]	South Africa
*Trypanosoma theileri*	MELSELE	[31]	Belgium
*Babesia bovis* c	Bbo-Gu	Unpublished	Venezuela
*Babesia bigemina* c	Bbig-Ya	Unpublished	Venezuela
*Anaplasma marginale* c	Am-Zu	Unpublished	Venezuela
*Theileria parva*	Katete	[32]	Zambia

^a^ Jimma zone, Chora Botor district, tsetse infested region.

^b^ Bale Zone and West Gojjam, tsetse free region.

^c^ DNA extracted from blood of an infected calf.

^d^ DNA extracted from blood of an infected sheep.

The analytical sensitivity of the *Tv*PRAC PCR was assessed on fivefold dilution series, ranging from 1 ng/µl down to 0.064 pg/µl, of DNA in water prepared from the Ethiopian *T. vivax* isolates 4337, 4338, Di and Fc. The same dilution series were used to assess the analytical sensitivity of ITS-1 PCR and of 18S PCR-RFLP [9]. The 18S PCR-RFLP is a semi-nested PCR, with 18S rDNA as target, followed by restriction enzyme digestion of the PCR product. The test was performed as described in [9]. The analytical sensitivity of *Tv*PRAC PCR was further evaluated on a tenfold serial dilution of live *T. vivax* trypanosomes in 0.5 ml volumes of naïve mouse blood ranging from 5.10^6^ down to 5 trypanosomes/ml.

To assess the agreement between *Tv*PRAC PCR on the one hand and ITS-1 PCR and 18S PCR-RFLP on the other hand for diagnosis of *T. vivax* infection, the three PCRs were conducted on 411 bovine blood specimens collected in Ethiopia between August 2010 and April 2011. Details on collection, microscopic examination, DNA extraction and results obtained with ITS-1 PCR are described elsewhere [21].

### Ethics statement

For the collection of blood specimens from bovine, camel, goat and mouse, ethical approval was obtained from the Ethics Committee for Veterinary Medicine of the Institute of Tropical Medicine Antwerp (EXT 2012-1 and EXT 2012-2). The human blood specimen was collected within a study approved by the Ethics Committee of the University Hospital of Antwerp (ITG 09415684). Written informed consent of the donor was obtained.

### Data analysis

SPSS version 20 was used to analyse the data and Kappa statistics [25] was applied to assess the degree of agreement between the three PCRs.

## Results

**Figure 1:**
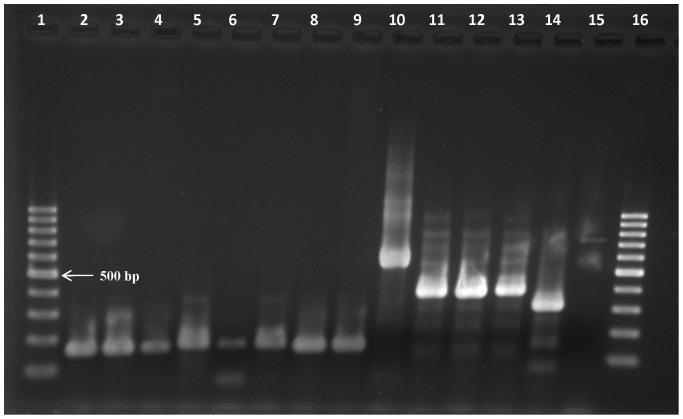
Amplicons generated with ITS-1 PCR on DNA of different trypanosome taxa at 1 ng/µl, except for lanes 4, 8 and 9 where the specimen contains also host DNA. Lanes 1 and 16 = 100 bp marker, lane 2 = *T. vivax* Y486, lane 3 = *T. vivax* Fc, lane 4 = *T. vivax* Sh, lane 5 = *T. vivax* 4337, lane 6 = *T. vivax* 4338, lane 7 = *T. vivax* Di, lanes 8 & 9 *T. vivax* LIEM 176, lane 10 = *T. congolense*, lane 11 = *T. brucei brucei*, lane 12 = *T. evansi*, lane 13 = *T. equiperdum*, lane 14 = *T. theileri*, lane 15 = negative extraction control.

ITS-1 PCR was performed using DNA of all the trypanosome species included in this study at a concentration of 1 ng/µl except for *T. vivax* Sh and LIEM 176, where the specimens contained 1 ng/µl of host and *T. vivax* DNA together. The ITS-1 PCR yielded amplicons with the expected length thus confirming the taxon identity (**Figure 1**).

**Figure 2:**
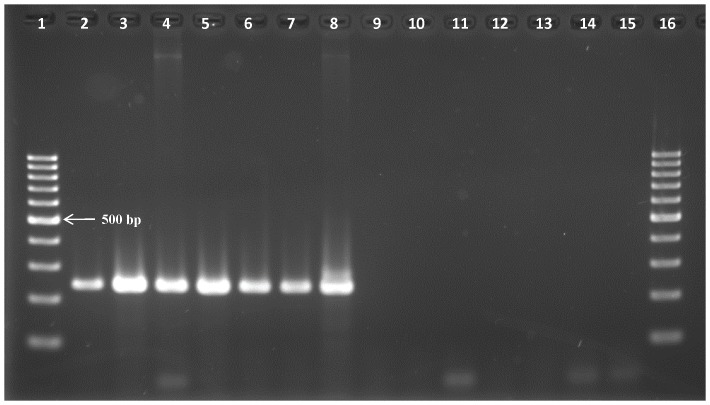
Amplicons generated with *Tv*PRAC PCR on DNA of different trypanosome taxa at 1 ng/µl, except for lanes 4, 8 and 9 where the specimen contains also host DNA. Lanes 1 and 16 = 100 bp marker, lane 2 = *T. vivax* Y486, lane 3 = *T. vivax* Fc, lane 4 = *T. vivax* Sh, lane 5 = *T. vivax* 4337, lane 6 = *T. vivax* 4338, lane 7 = *T. vivax* Di, lanes 8 & 9 *T. vivax* LIEM 176, lane 10 = *T. congolense*, lane 11 = *T. brucei brucei*, lane 12 = *T. evansi*, lane 13 = *T. equiperdum*, lane 14 = *T. theileri*, lane 15 = negative extraction control.

**Figure 3:**
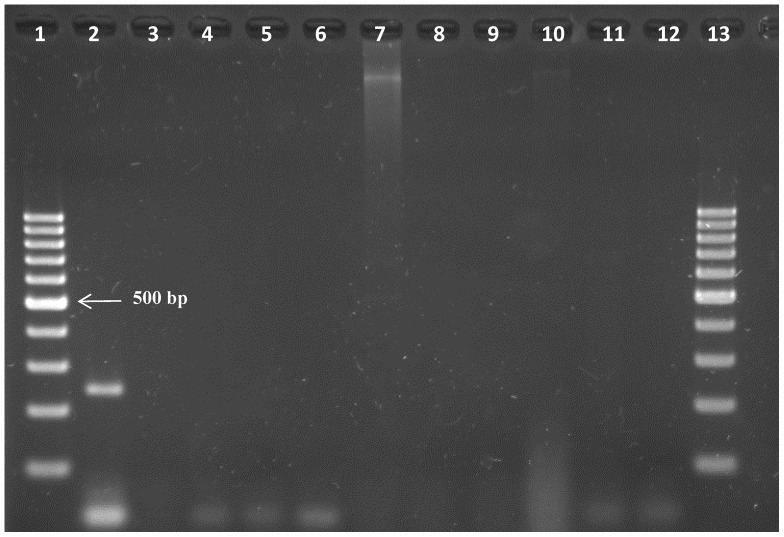
Amplicons generated with *Tv*PRAC PCR on DNA of non-target species at 1 ng/µl. Lanes 1 & 13 = 100 bp marker, lane 2 = positive control (*T. vivax* Y486), lane 3 = *Theileria parva*, lane 4 = *Anaplasma marginale*, lane 5 = *Babesia bovis*, lane 6 = *Babesia bigemina*, lane 7 = bovine, lane 8 = goat, lane 9 = camel, lane 10 = mouse, lane 11 = human, lane 12 =  negative extraction control.

The specificity of the *Tv*PRAC PCR was tested on the same trypanosome DNA collection, on DNA of other haemoparasites of cattle, and on host DNA of mammals and human. Only with *T. vivax* DNA, irrespective of the strain, *Tv*PRAC PCR yielded the expected 239 bp amplicon (**Figure 2**). Note that lane 9 remained negative since the PCR reaction contained <1 µl of template DNA. No amplicons were generated with non-target DNA (**Figure 3**).

**Figure 4:**
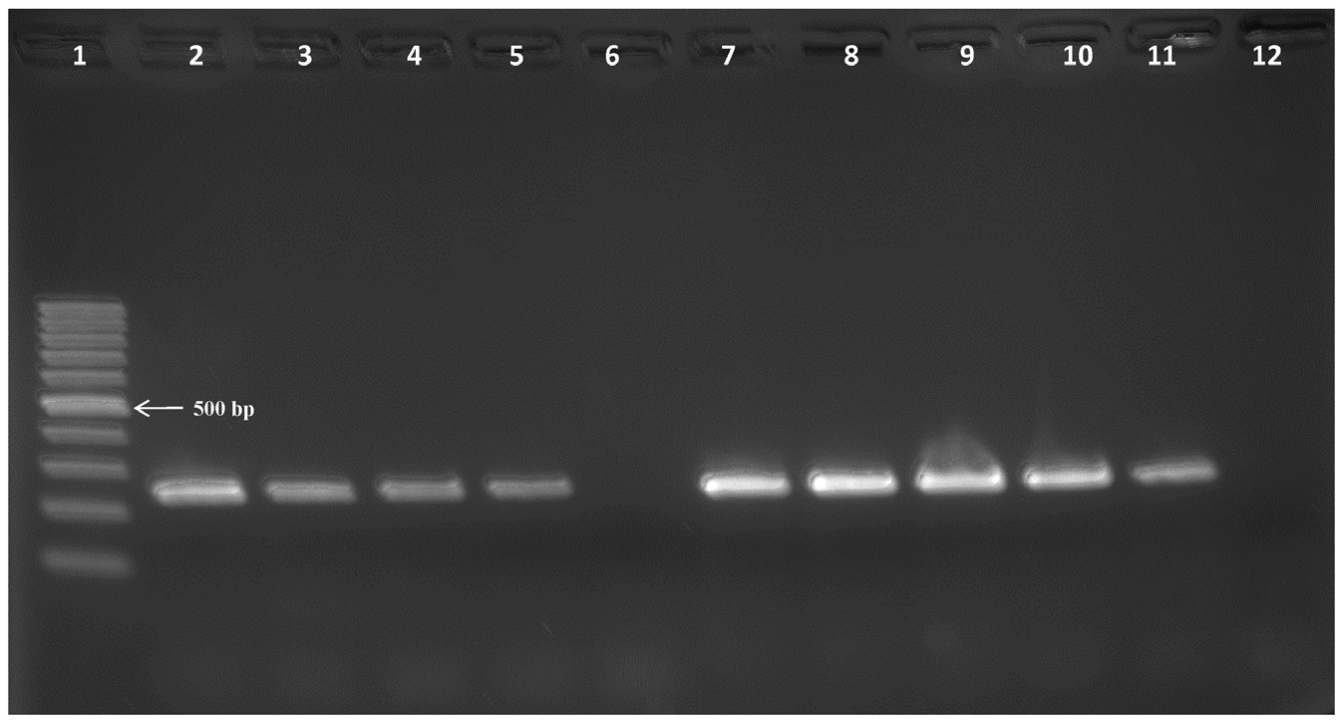
Assessment of the lower detection limit of *Tv*PRAC PCR on a fivefold DNA dilution series in water. Lane 1 = 100 bp marker, lanes 2 to 6: *T. vivax* 4337 DNA at 1000, 200, 40, 8, 1.6 pg/µl, lanes 7 to 11: *T. vivax* Fc DNA at 1000, 200, 40, 8, 1.6 pg/µl, 12 = negative extraction control.

**Figure 5:**
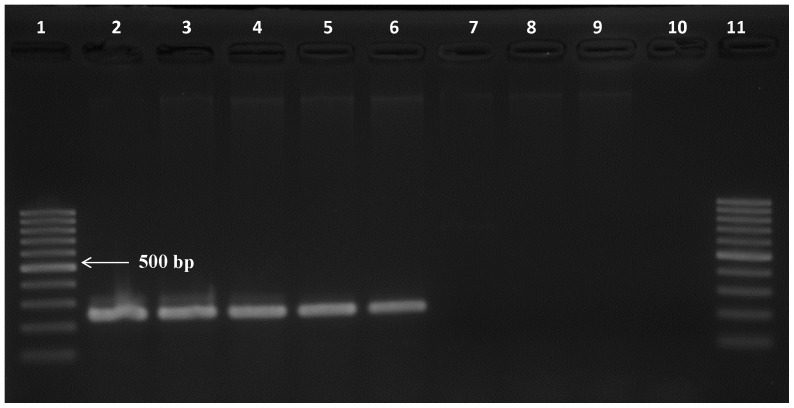
Assessment of the lower detection limit of *Tv*PRAC PCR on a tenfold dilution series of parasites in mouse blood. Lane 1 = 100 bp marker, lanes 2 to 8: *T. vivax* Y486 parasites at 5.10^6^ down to 5 trypanosomes/ml, lane 9 =  naïve mouse blood, lane 10 =  negative extraction control, lane 11 = .100 bp marker.

The analytical sensitivities of the *Tv*PRAC PCR, ITS-1 PCR and 18S PCR-RFLP, were assessed on fivefold dilution series of *T. vivax* DNA in water (**Figure 4** **and** **Table 2**). As shown in **Figure 4** for *T. vivax* 4337 and Fc, the lower detection limit of *Tv*PRAC PCR ranges from 8 pg/µl to 1.6 pg/µl depending on the strain. From Table 2, it appears that for three isolates, 18S PCR-RFLP remained negative at 1000 pg/µl. Depending on the strain, the *Tv*PRAC PCR showed either a lower or higher detection limit than ITS-1 PCR. With *T. vivax* Y486 that grows in mice, we were able to assess also the analytical sensitivity of *Tv*PRAC on DNA extracted from blood containing live parasites and found that it was 500 parasites/ml (**Figure 5**).

**Table 2:** Lower detection limits expressed as pg/µl of *Tv*PRAC PCR, ITS-1 PCR and 18S PCR-RFLP assessed on DNA dilutions of four Ethiopian *T. vivax* isolates.

	Lower detection limit (pg/µl)		
T. vivax isolates	TvPRAC PCR	ITS-1 PCR	18S PCR-RFLP
4337	8	1.6	>1000
4338	40	1000	>1000
Di	8	40	>1000
Fc	1.6	0.32	8

**Figure 6:**
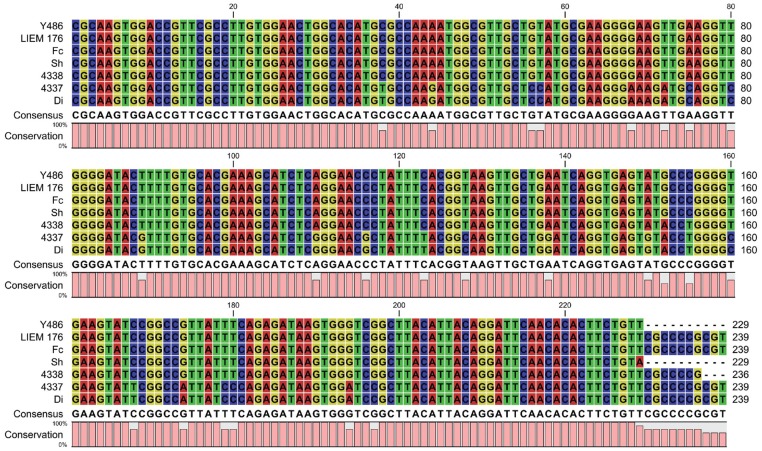
Alignment of the *Tv*PRAC PCR amplicon sequences of the different *T. vivax* isolates with *T. vivax* ILRAD 1392 as reference sequence in GenBank.

The *Tv*PRAC PCR products from the different *T. vivax* strains were sequenced, directly from the PCR product (4337, 4338, Fc, Sh, Di, Y486) or after cloning into *E. coli* (LIEM 176). Alignment with the ILRAD 1392 sequence published in GenBank reveals that the amplicon sequences of Y486, the two Ethiopian isolates from tsetse free region (Fc and Sh), and the Venezuelan isolate LIEM 176, are almost identical to each other and to the corresponding sequence of ILRAD 1392, as shown in **Figure 6**. There are sequence variations among the Ethiopian *T. vivax* isolates from tsetse infested regions (4338, 4337 and Di). The amplicons of strains 4338, 4337 and Di are respectively 99%, 90% and 90% similar to the corresponding sequence of ILRAD 1392.

The agreement between *Tv*PRAC PCR and ITS-1 PCR and 18S PCR-RFLP, assessed on 411 specimens from cattle under natural infection conditions, is represented in **Table 3**. *Tv*PRAC PCR detects *T. vivax* infection in 8.3% of the tested specimens compared to 22.6% and 6.1% detected with ITS-1 PCR and 18S PCR-RFLP respectively. Here it is important to note that 9 specimens were positive in *Tv*PRAC PCR but negative in ITS-1 PCR. The degree of agreement between the *Tv*PRAC PCR and the other two PCRs is fair with K = 0.4 between *Tv*PRAC PCR and 18S PCR-RFLP and K = 0.3 between *Tv*PRAC PCR and ITS-1 PCR.

**Table 3:** Cross tabulation of results obtained with the three PCRs on 411 specimens from cattle under natural infection conditions.

Test		*Tv*PRAC PCR					
		Positive		Negative		Total	
		n	%	n	%	N	%
18S PCR-RFLP	Positive*	13	3.2	12	2.9	25	6.1
	Negative	21	5.1	365	88.8	386	93.9
ITS-1 PCR	Positive*	25	6.1	68	16.5	93	22.6
	Negative	9	2.2	309	75.2	318	77.4
Total		34	8.3	377	91.7	411	

for the *T. vivax*-specific band.

## Discussion

This study aimed to develop a PCR that is able to unequivocally identify *T. vivax* from diverse geographical origin with particular interest in strains circulating in East Africa. The *Tv*PRAC PCR is based on a *T. vivax* specific proline racemase gene described in the West African *T. vivax* ILRAD 1392 strain [24]. In contrast with other species-specific molecular tests, the *Tv*PRAC PCR developed in this study detected *T.vivax* from Nigeria (*T. vivax* Y486), from Venezuela (LIEM 176) as well as from Ethiopia (4337, 4338, Fc, Di, Sh). Furthermore, alignment of *Tv*PRAC PCR amplicons with the corresponding sequence of *T. vivax* ILRAD 1392 in GenBank showed none or limited sequence variations among the different strains that were tested. These findings make the *Tv*PRAC PCR a valuable candidate as a molecular diagnostic tool for *T. vivax* infection worldwide. The specificity of the primers proved excellent since *Tv*PRAC PCR remained negative on DNA from other African trypanosome taxa and from other cattle infecting pathogens like *Babesia bovis*, *B. bigemina*, *Theileria parva* and *Anaplasma marginale*. *Tv*PRAC PCR remained also negative with *T. cruzi* (data not shown). In addition, DNA from natural and laboratory host species as well as from man was not amplified, thus eliminating the possibility of false positive results due to contamination with host or manipulator DNA.

Using purified DNA in water, the analytical sensitivity of the *Tv*PRAC PCR varied among the Ethiopian strains with a factor of 25 from 1.6 to 40 pg/µl while with the ITS-1 PCR, this parameter varied with a factor of 3125 from 0.32 to 1000 pg/µl. With the 18S PCR-RFLP three out of four strains remained even negative at the highest DNA concentration of 1000 pg/µl. The latter finding is consistent with the low number of *T. vivax* infections (6.1%) detected with the 18S PCR-RFLP in the bovine blood collection in this study. Up to now, the variation of analytical sensitivity of the *Tv*PRAC PCR and of the ITS-1 PCR among the tested strains remains unexplained. It might be attributed to different copy numbers of the target sequences in the respective genomes although the proline racemase gene has been described as a single copy gene in the *T. vivax* ILRAD 1392 strain [24]. Another possibility could be small mismatches between the primers and the target sequences, both in the *Tv*PRAC PCR and the ITS-1 PCR. The different copy number of the proline racemase gene and the ITS-1 sequences most probably underlies the differences in observed prevalence in the bovine blood collection (8.3% with *Tv*PRAC PCR and 22.6% with ITS-1 PCR).

Depending on the situation, we believe that the combined application of both ITS-1 PCR and *Tv*PRAC PCR may take advantage of the characteristics of both tests. ITS-1 PCR can detect multiple species, including mixed infections, in one single reaction and has a high diagnostic sensitivity, although in some instances, it will not detect *T. vivax* infections that are detectable with *Tv*PRAC PCR. The latter test, on the other hand, has the advantage that it is species specific and that it reacts with *T. vivax* strains from diverse geographical origin with a lower detection limit of about 500 trypanosomes/ml. Further improvement in primer design might increase the analytical sensitivity of the *Tv*PRAC PCR which may be very important for studying the prevalence of *T. vivax* in areas where tsetse do not naturally occur or where this vector has been eradicated.
